# Public health round-up

**DOI:** 10.2471/BLT.21.010521

**Published:** 2021-05-01

**Delivering COVID-19 vaccines worldwide**

A manager in charge of COVID-19 vaccination checks vaccination cards and vials at the Paropakar Maternity and Women’s Hospital in Kathmandu, Nepal. Nepal is one of 102 economies to have received a total of 38 million doses of COVID-19 vaccines through the COVAX facility between 24 February and 8 April.

**Figure:**
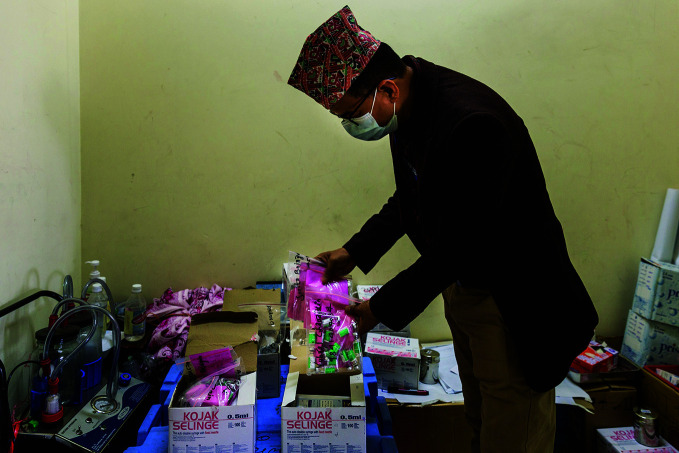
WHO/Blink Media/Uma Bista

## COVAX delivers

COVAX delivered more than 38 million doses of coronavirus disease 2019 (COVID-19) vaccine in the six weeks following its first deliveries to Ghana in February. As of 8 April, COVAX – the vaccines pillar of the Access to COVID-19 Tools (ACT) Accelerator – had delivered vaccine to 102 economies across six continents. Some 61 of those economies were among the 92 lower-income economies receiving vaccines funded through the Gavi COVAX Advance Market Commitment.

The vaccines were supplied by AstraZeneca, Pfizer-BioNTech and the Serum Institute of India.

Despite supply issues experienced in March and April, COVAX expects to deliver doses to all participating economies that have requested vaccines in the first half of 2021.

Dr Seth Berkley, CEO of Gavi, the Vaccine Alliance, said that while COVAX may be on track to meet delivery goals, this is no time for complacency. “Our efforts to rapidly accelerate the volume of doses depend on the continued support of governments and vaccine manufacturers,” he said.

https://bit.ly/3tiVFun

## AstraZeneca vaccine risk/benefit

The COVID-19 subcommittee of the World Health Organization (WHO) Global Advisory Committee on Vaccine Safety recommended that the rare cases of blood clots with low platelets following vaccination with the AstraZeneca COVID-19 vaccine be assessed against the risk of deaths from COVID-19 disease. As of 7 April, an estimated 2.86 million people had died of the disease worldwide.

The subcommittee reviewed the latest information from the European Medicines Agency at its meeting on 7 April along with information from other agencies, including the Medicines & Healthcare Products Regulatory Agency of the United Kingdom of Great Britain and Northern Ireland.

The subcommittee called for further studies to fully understand the potential risks associated with the vaccine and recommended that people experiencing severe symptoms from around four to 20 days following vaccination – including chest pain, leg swelling, persistent abdominal pain, severe and persistent headaches or blurred vision – seek urgent medical attention.

https://bit.ly/3mIvM4H

## COVID-19 origins

A joint international team led by WHO tasked with identifying the source of the COVID-19 virus and the route of introduction to the human population reached no firm conclusion. Published on 30 March, the report covered the team’s 14 January–10 February field visit to Wuhan, China.

The team examined four possibilities for introduction and assessed the likelihood of each. Direct zoonotic transmission to humans was assessed to be possible-to-likely; introduction through an intermediate host followed by transmission to humans was assessed to be likely to very likely; introduction through the cold food chain was assessed to be possible; and introduction through a laboratory incident was assessed to be extremely unlikely.

In conclusion, the team called for continued scientific collaboration to work towards tracing the origins of COVID-19. WHO Director-General Tedros Adhanom Ghebreyesus also called for further studies, saying, “As far as WHO is concerned, all hypotheses remain on the table.”

https://bit.ly/3db6CZk

## Continuing HIV services in the pandemic

The benefits of continuing to provide human immunodeficiency virus (HIV) services during the pandemic far outweigh the risk of additional COVID-19-related deaths associated with the provision of those services. This is the main conclusion of an analysis published by the Joint United Nations Programme on HIV/AIDS and WHO on 14 April.

Derived from mathematical modelling designed to establish the benefits of continuing HIV services compared to the potential harm of additional COVID-19 transmission, the analysis shows that maintaining HIV services would avert between 19 and 146 deaths related to acquired immunodeficiency syndrome per 10 000 people over a 50-year time horizon, while the additional COVID-19-related deaths from exposures related to HIV services would be 0.002 to 0.15 per 10 000 people.

“This work shows that taking the longer view, the benefits of continuing key HIV services are far larger than the risks of additional COVID-19 transmission,” said Meg Doherty, Director of WHO’s Global HIV, Hepatitis and Sexually Transmitted Infections Programmes. “Innovative and safe delivery of services must continue as the pandemic is brought under control.”

https://bit.ly/3wSTRue

## Sales of live wild mammals

WHO, the World Organisation for Animal Health and the United Nations Environment Programme issued interim guidance on reducing public health risks associated with the sale of live wild mammals in traditional food markets. Issued on 13 April, the guidance calls for the suspension of sales of captured live wild mammals in food markets as an emergency measure.

Animals are the source of more than 70% of all emerging infectious diseases in humans, many of which are caused by novel viruses. 

https://bit.ly/3sfw2ck

## Global Diabetes Compact

WHO launched a Global Diabetes Compact aimed at boosting efforts to prevent diabetes and bring treatment to all who need it, 100 years after the discovery of insulin.

The Compact will bring together key stakeholders from the public and private sectors, and, critically, people who live with diabetes, around a common agenda focused on increasing access to diabetes diagnostic tools and medicines, particularly insulin, in low- and middle-income countries.

Global coverage targets for diabetes care will be established and a “global price tag” will quantify the costs and benefits of meeting the targets.

The Compact was launched on 14 April at a Global Diabetes Summit co-hosted by WHO and the Government of Canada, with the support of the University of Toronto.

https://bit.ly/3e07bV4

## Calls for pandemic treaty

Heads of government and international agencies called for the drawing up of an international pandemic treaty, signalling the need for high-level political action to protect the world from future health crises.

In an editorial published on 30 March and widely disseminated through the international press, the leaders stated that no single government or multilateral agency can address the threat of future pandemics alone and that the global community must come together to predict, prevent, detect, assess and effectively respond to pandemics in coordination.

The main goal of a new international treaty for pandemic preparedness and response would be to foster a comprehensive, multisectoral approach to strengthen national, regional and global capacities and resilience to future pandemics.

https://bit.ly/3a9UMwF

**Cover photo**

A health worker makes an entry into a register in the Badegaon Primary Health Care Center in the Lalitpur District of Nepal.

**Figure:**
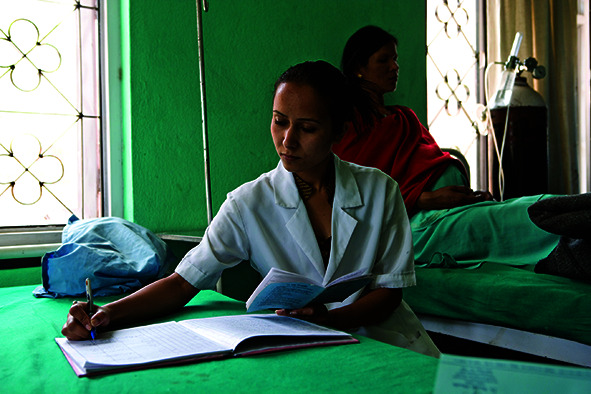
WHO/Christopher Black

## Pandemic impacts tuberculosis care

An estimated 1.4 million fewer people received care for tuberculosis (TB) in 2020 than in 2019. A new report based on preliminary data from 84 countries published by WHO on 22 March reveals that 4.9 million cases (provisional) were reported in 2020, compared with 6.3 million reported for 2019 – a 21% drop. The biggest reductions in reported cases were registered in India, Indonesia, the Philippines and South Africa.

Modelling of the impact of reductions in TB detection and care in 2020 on TB mortality indicated that a half a million excess TB deaths could result, setting the world back a decade, to the level of TB mortality recorded in 2010.

https://bit.ly/3mHLT2g

## Taxing tobacco

An estimated US$ 1.4 trillion is lost in health expenditures and lost productivity due to tobacco use every year. This is according to a new manual on tobacco tax policy and administration released by WHO.

Launched on 12 April, the manual details strategies for effective tobacco tax policy development, design, implementation and administration, incorporating the latest developments in science, technology and policy, as well as providing illustrative recent examples from a variety of countries.

Increases in excise taxes that drive up tobacco product prices have consistently proven to be the most effective mechanism for reducing tobacco consumption. Despite this, tobacco tax increases remain the least implemented policy in the package of effective tobacco control policies globally. In 2018 only 38 countries, covering 14% of the global population, had tobacco taxes judged to be sufficiently high.

The manual is designed to support governments in the development of tobacco taxation policy, facilitating the achievement of their health and revenue objectives while also supporting their overall development strategies.

https://bit.ly/3mJYGBa

**Looking ahead**

06–07 May. Leadership Summit on Tobacco Control. https://bit.ly/3sZpFuS

13 May. WHO Health for All Film Festival Awards. https://bit.ly/3v0316t

17–23 May. 6th UN Global Road Safety Week. https://bit.ly/3wBm88w

